# Complexity Synchronization of Energy Volatility Monotonous Persistence Duration Dynamics

**DOI:** 10.3390/e21101018

**Published:** 2019-10-20

**Authors:** Linlu Jia, Jinchuan Ke, Jun Wang

**Affiliations:** 1 School of Economics and Management, Beijing Jiaotong University, Beijing 100044, China; 2 School of Science, Beijing Jiaotong University, Beijing 100044, China

**Keywords:** volatility monotonous persistence duration, statistical and nonlinear analysis, complexity synchronization, cross-fuzzy entropy, complexity-invariant distance, ensemble empirical mode decomposition

## Abstract

A new concept named volatility monotonous persistence duration (VMPD) dynamics is introduced into the research of energy markets, in an attempt to describe nonlinear fluctuation behaviors from a new perspective. The VMPD sequence unites the maximum fluctuation difference and the continuous variation length, which is regarded as a novel indicator to evaluate risks and optimize portfolios. Further, two main aspects of statistical and nonlinear empirical research on the energy VMPD sequence are observed: probability distribution and autocorrelation behavior. Moreover, a new nonlinear method named the cross complexity-invariant distance (CID) FuzzyEn (CCF) which is composed of cross-fuzzy entropy and complexity-invariant distance is firstly proposed to study the complexity synchronization properties of returns and VMPD series for seven representative energy items. We also apply the ensemble empirical mode decomposition (EEMD) to resolve returns and VMPD sequence into the intrinsic mode functions, and the degree that they follow the synchronization features of the initial sequence is investigated.

## 1. Introduction

Financial markets have a large number of participants. With the continuous financial innovation, more and new financial instruments have made investors have more investment choices. However, financial markets can bring higher returns to investors, they also face corresponding risks [1]. Especially with the globalization of the economy, notional governments are opening up security markets, and financial markets of various countries began to interact and influence each other. In addition to the emergence of various new derivatives, more and more risks appeared in security markets. Therefore, the importance of studying the price fluctuation of security markets became more and more obvious and became one of the important issues in financial market researches. The Security market is often considered as a complex system exhibiting many nonlinear and complex characteristics [2,3]. As an important part of the security market, the relevant characteristics of energy market have recently attracted widespread attention. Oil has a major impact on economic and political developments, especially on international energy markets [4]. For the past few years, a lot of valuable researches have been conducted on oil prices, including relationship between oil prices and stock markets from different regions [5], multiscale entropy analysis of crude oil price dynamics [6], and forecasting of crude oil price with neural networks [7], etc. Among them, the exploration of return volatility dynamic is a significant subject for investors and decision makers, because it is a matter of great account in evaluating risks, modeling market dynamics and enabling portfolios to be optimized [8,9,10,11,12,13,14,15,16,17,18,19]. Specifically, investors in energy markets are always faced with the problem of choosing the optimal portfolios, while unpredictable volatility behaviors are often the main investment risks. Recently, various novel means for exploring the volatility behaviors have been developed from different perspectives [20,21,22,23,24,25,26,27,28]. For instance, realized volatility [20,21], an often-used measure with a simple definition but important, is the sample variance of high frequency historical return data in a fixed interval. Wang et al. [21] studied statistical properties of realized volatility of crude oil futures and Henry-Hub natural gas futures from the New York Mercantile Exchange (NYMEX) based on daily return data. Furthermore, the return interval is applied in the daily fluctuations of the price changes that are above a certain threshold. Xie et al. [23] applied the recurrence interval to daily return volatility of four energy futures and studied the scaling behaviors and memory effect behaviors. Moreover, Yang et al. [24] put forward another nonparametric volatility measure called return volatility duration, which is used to describe the minimum duration when the prospective volatility intensity is bigger or smaller than the current volatility intensity at each time. The idea of volatility duration of return series has been introduced into the complex analysis study of energy futures and spot data by Niu and Wang [25]. Inspired by the above measure approaches, in this paper, a new idea of volatility monotonous persistence duration (VMPD) time sequence is introduced into energy markets to describe the energy security volatility behaviors from a new perspective. Compared to the previous measure approaches, the VMPD on the one hand considers the continuous variation length (growth/descent), on the other hand takes into account the maximum fluctuation difference. Among them, the continuous variation length represents the duration of the monotonous uptrend or downward trend of return volatility. In financial markets, the monotonous upward trend (or monotonous upward trend) means rising (or decreasing) return volatility, which may influence investors’ investment risk. The maximum fluctuation difference is used to describe the change in the intensity of return volatility over the monotonous trend, which is also important for investors to make investment decisions. At the times of the same continuous variation length, the magnitudes of the maximum fluctuation difference may be different, which represents different investment risks. By combining the continuous variation length and the maximum fluctuation difference, the VMPD can reveal more information about price volatility, and be regarded as a novel indicator to reveal the risk status of financial markets. The larger the maximum fluctuation difference is and the larger the continuous variation length is, the larger the VMPD is. Investors can better judge risk levels, choose the appropriate portfolio and make decisions depending on the VMPD. Moreover, the VMPD provides financial researchers with a new perspective on energy market volatility behaviors, thus providing some recommendations for the establishment of national macroeconomic policies. This new proposed concept is brought in the research of seven kinds of energy data including futures and spots from different energy markets.

We investigate fluctuation properties and complexity synchronization of energy VMPD sequences. Recent empirical studies on security time series indicate that there exist some common statistical and nonlinear features in different security markets, for instance including excess kurtosis, fat tails, absence of autocorrelation, multifractal nature, complexity behaviors [23,29,30,31,32]. Firstly, we observe some important statistical characteristics for the energy VMPD sequences: probability distribution, power-law behaviors, and autocorrelation behavior. Further, we study the complexity synchronization of VMPD series for different energy items. Financial market is a complex dynamic system, and its fluctuations are often accompanied by nonlinear and dynamic characteristics, which cannot be reproduced well by many traditional measurement methods. Synchronization provides insight into the fundamentals of interaction mechanism between systems, and it is an important concept that is widely used in many fields. Recently, synchronization has been studied by various methods, such as projection synchronization [33] and generalized synchronization [34]. The price formation of economic market is the result of the complicated action of many factors, and entropy is an index of pattern diversity in time series. It is a worthy way to study the synchronization from the perspective of entropy. The concept of entropy has been widely used as a measurement of complexity [35,36,37,38,39,40,41,42,43,44,45]. Fuzzy entropy (FuzzyEn) [39,40] is a reflection of similarity of time series evolved from approximate entropy (ApEn) [38] and sample entropy (SampEn) [37]. Compared to ApEn and SampEn, FuzzyEn has many advantages. Furthermore, Chen et al. [43] extended FuzzyEn method to cross-fuzzy entropy in order to compare two different time series, and it is relatively consistent and less dependent on record length in measuring regularity and asynchrony of time series. The idea of using entropy concepts to analyze time series of financial and economical markets is widely accepted. Darbellay and Wuertz [44] analyzed several sets of financial time series and proved the validity of the entropy method. Risso [45] used entropy to quantify market efficiency of the stock markets. Another complexity analysis method is complexity-invariant distance (CID) [46,47], which has been applied in measuring comparability between two time sequences. On account of the above considerations, we put forward a new method called the cross CID FuzzyEn (CCF), which is composed of cross-fuzzy entropy and complexity-invariant distance to calculate the synchronization for two time series of the same length. Introducing the CCF analysis to seven representative energy items, the synchronization properties of returns and VMPD series are compared an analyzed. Then we apply the ensemble empirical mode decomposition (EEMD) [48,49,50] to resolve returns and VMPD series into the intrinsic mode functions, and the degree that they follow the synchronization features of initial sequence is investigated.

The main contributions of this paper include the following aspects. One is that a new idea of volatility monotonous persistence duration (VMPD) time sequence is put forward to investigate the energy market fluctuation behaviors from a new perspective. The other is that a new nonlinear estimate method – the cross CID FuzzyEn (CCF) composed of cross-fuzzy entropy and complexity-invariant distance is put forward, and the CCF analysis is applied for seven actual representative energy items to investigate the synchronization features of returns and VMPD series. Lastly, the EEMD algorithm is used to resolve the returns and VMPD sequence into the intrinsic mode functions to further investigate the corresponding synchronization behaviors. This present work can provide new insights into the price volatility dynamics.

The layout of this paper is detailed below. Section 2 shows the definition of the VMPD series. Section 3 illustrates the data sets that are adapted in this work. In Section 4, we observe the power-law distribution and the autocorrelation characteristics for the energy VMPD sequence. Section 5 investigates the complexity synchronization of VMPD series and intrinsic mode functions (decomposed by VMPD series) for different energy items. Finally, Section 6 gives the conclusions.

## 2. Mathematical Concept Description of VMPD Series

**Figure 1:**
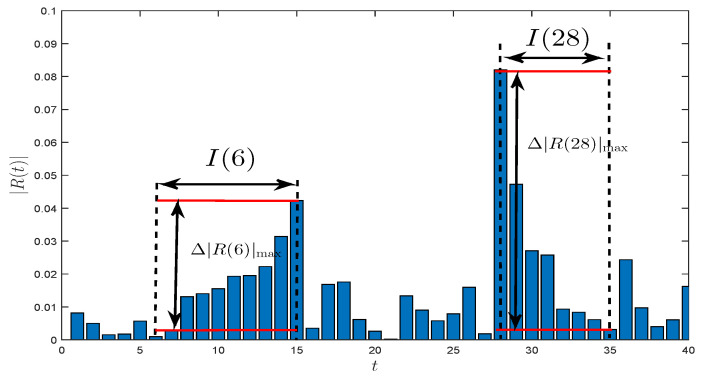
Illustrations of I(t) and ${\Delta |R\left(t\right)|}_{\max}$ for crude oil WTI futures.

We here introduce a new kind of statistic called volatility monotonous persistence duration (VMPD) series {V(t)} for return series {R(t)} (t∈{1,2,⋯,N}) into the energy markets to study the security fluctuation with a novel perspective, and the composition of the new sequence is shown below. Use P(t) to represent the daily price of an energy item for every business day *t*, and let R(t) represent the return of the prices which is calculated by R(t)=lnP(t)−lnP(t−1), and |R(t)| be the corresponding absolute return. At each trading day *t*, we will consider the absolute return of the following day |R(t+1)|:

(i) If |R(t+1)|<|R(t)|, the volatility sequence is thought to be locally falling at day *t*. Then we define the length of continuous growth I(t) at day *t*, namely
  (1) I(t)=max{τ:|R(i+1)|<|R(i)|,forallt≤i≤τ}.
(ii) If |R(t+1)|>|R(t)|, the volatility sequence is thought to be locally rising at day *t*. Then we have define the length of sustained descent I(t) at day *t*, namely
  (2) I(t)=max{τ:|R(i+1)|>|R(i)|,forallt≤i≤τ}.
(iii) Specifically, when |R(t+1)|=|R(t)|, we assume that I(t) = 0.
(iv) Let ${\Delta |R\left(t\right)|}_{\max}=\left|R\left(t+I\left(t\right)\right)\right|-\left|R\left(t\right)\right|$ denote the maximum fluctuation difference, in other words, it is also the largest growth or decline during the continuous variation length I(t). Schematic diagrams of I(t) and ${\Delta |R\left(t\right)|}_{\max}$ are shown in Figure 1.
(v) Then, the VMPD sequence {V(t)} is defined by:
  (3) $$\;V\left(t\right)=\left\{\begin{array}{c} +{\Delta |R\left(t\right)|}_{\max}\times I\left(t\right),\;\mathrm{if}\;|R(t+1)|>|R(t)|\\ -{\Delta |R\left(t\right)|}_{\max}\times I\left(t\right),\;\mathrm{if}\;|R(t+1)|<|R(t)| \end{array}.\right.$$

It’s worth noting that when I(t) = 0 or |R(t+1)|=|R(t)|, we define V(t) = 0. Thus, V(t) unites the maximum increase/decrease ${\Delta |R\left(t\right)|}_{\max}$ and the continuous growth/descent length I(t), which describes the volatility monotonous persistence duration at each trading day *t*. The proposed idea provides a novel study approach and research perspective on fluctuation behaviors of energy markets. Figure 1 well illustrates the VMPD sequence of the crude oil WTI futures in 40 trading days.

## 3. Basic Statistical Description of Data Sets

**Figure 2:**
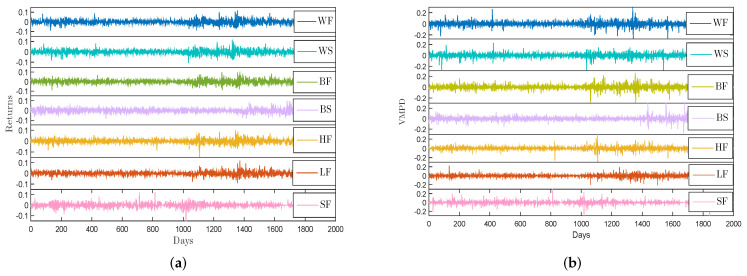
(**a**) The return series R(t) of energy futures and spot data; (**b**) The VMPD series V(t) of energy futures and spot data.

In this paper, seven daily closing data of prices for energy items from three energy markets are considered: crude oil WTI futures, crude oil WTI spot, heating oil futures (they are from New York Mercantile Exchange); together with crude oil Brent futures, crude oil Brent spot, London gas oil futures (they are from Intercontinental Exchange); and Shanghai fuel oil futures (it is from Shanghai Futures Exchange). These abbreviations for energy data are listed in the previous section of the article. The unit of price for crude oil is barrel, the unit of price for fuel oil is gallon, and the unit of price for gasoline is metric ton. The lengths of the seven data sets are the same, from January 2010 to July 2018 with about 2001 data points respectively. This choice was made for the following reasons. Firstly, as an important part of the energy market, energy futures market has the advantages of low transaction cost and no restriction on short selling. It is often able to reflect the price information of the energy market in a timely and accurate manner. However, the high leverage of energy futures market makes the energy futures market face greater risks than the spot market. Therefore, the study of oil futures and spot price fluctuations is of great theoretical value and practical significance for preventing the risk of energy price fluctuations and promoting the stable development of the energy market. Secondly, the energy futures market is based on oil futures products, and oil futures has the functions of price discovery, hedging and risk avoidance. The Brent crude oil futures in the north sea and the west Texas light crude oil futures in the United States are the benchmark for international oil pricing, while the Shanghai fuel oil futures is currently an important product in China. Other energy products selected in this paper are also based on the consideration that they are important indicators of their respective energy markets. Figure 2 shows returns R(t) and VMPD series V(t) for these different energy data. For the crude oil WTI futures and the crude oil Brent futures, the shapes of R(t) and V(t) are exactly alike with the shapes of the series for their spots. The extreme values of VMPD sequence are concentrated in the interval [−0.2,0.2].

From Table 1, it can be seen that the average value of each energy item is close to 0, the kurtosis coefficient is greater than 3, and the skewness coefficient is significantly not 0. This indicates that these different energy futures and spots are not subject to normal distribution, and have characteristics of excess kurtosis and fat tail [51]. The results of K-S statistics also prove this conclusion. In the K-S test of this paper, the significance level is 5%. In Table 1, we can see that the null hypothesis is rejected, because all the test logic values H=1 are returned and the value of JB statistics of each data group is higher than the critical value 5.96 under the significance level of 5%, indicating that the test data does not conform to the normal distribution. In addition, it can be seen from the standard deviation of these seven time sequence that the New York Mercantile Exchange has the strongest degree of fluctuation, while the Intercontinental Exchange is the weakest.

## 4. Statistical and Nonlinear Behaviors of Return Series and VMPD Series

Recent empirical studies on security time series indicate that there exist some common features in different security markets [23,29], including excess kurtosis, fat tails [30], and absence of autocorrelation [32]. In this section, we use several statistical and nonlinear approaches to investigate if the proposed VMPD series V(t) of the energy data sets mentioned above have these properties.

### 4.1. Probability Density Distribution

**Figure 3:**
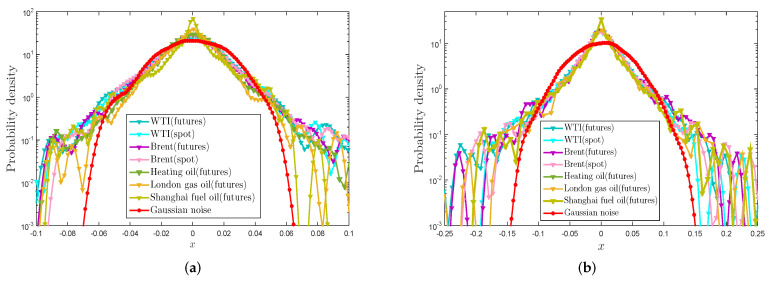
(**a**) Logarithmic plots of probability density functions by kernel density estimation of return series R(t); (**b**) Logarithmic plots of probability density functions by kernel density estimation of VMPD series V(t).

In Section 3, we verified that the return series R(t) and the proposed VMPD series V(t) for the energy items have characteristics of excess kurtosis and fat tail by using some descriptive statistics and K-S test. In this part, we will further investigate the probability density distributions (PDF) for R(t) and V(t) with the help of intuitive graphs. Logarithmic plots of probability density distributions for these time series in comparison with the Gaussian distribution are presented in Figure 3. The patterns obtained by kernel density estimation reveal that R(t) and V(t) for the energy items deviate from the Gaussian and exhibit excess kurtosis and fat-tail distributions.

### 4.2. Power-law Behaviors

According to the above research, since the R(t) and V(t) sequences do not obey the normal distribution, while Gabaix et al. [52] show that the power-law distribution is used to describe distribution of tail data of real financial market volatility related time series, such as the volatility of stock price and volume, and the formula f(x) = $\beta x^{\gamma }$ can fit the tail distribution well, where γ represents the power-law exponent and β is a constant [53]. We will test if tails of absolute return series |R(t)| and absolute VMPD series |V(t)| obey the power-law theory.

**Figure 4:**
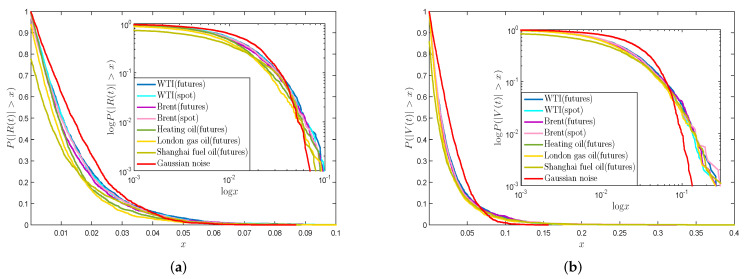
(**a**) Plots and log-log plots of cumulative distributions of absolute return series |R(t)|; (**b**) Plots and log-log plots of cumulative distributions of absolute VMPD series |V(t)|.

Figure 4a,b display the plots and log-log plots of cumulative distributions of absolute return series |R(t)| and absolute VMPD series |V(t)|. Figure 4 exhibits that |R(t)| and |V(t)| series for the energy data from diverse markets have parallel tracks of cumulative distributions. It is clear that for both |R(t)| and |V(t)| series of these analyzed energy data are all below the normal distribution, which illustrate that they have fat tails. Moreover, the tail of each curve is close to a straight line, which indicates that the tails of all data obey the power-law distribution. All time series are fitted with the last 10% data, and Table 2 shows the estimation values of γ and β. From Table 2, we can see that the values of exponent parameter γ are basically around −3, and the absolute γ value for |R(t)| series is bigger than that for |V(t)| series in general. It is noted that the *R*-square values are all over 0.95, which shows that the tails of energy |R(t)| and |V(t)| series can be fitted by the power-law formula.

### 4.3. Autocorrelation Analysis

In this part, we adopt the autocorrelation approach [54] to explore the possible long-term correlation of return series R(t) and proposed VMPD series V(t) for the energy items. The autocorrelation function is defined as follows [55]

(4) $$A\left(X_{t},k\right)=\sum_{i=k+1}^{N}\left(\left(X_{i}-\bar{X}\right)\left(X_{i-k}-\bar{X}\right)\right)/\sum_{i=1}^{N}{\left(X_{i}-\bar{X}\right)}^{2},\;k=0,1,\cdots ,N-1$$

where $X_{t}$ is the returns or the VMPD series of energy items, $\bar{X}$ is the mean of $X_{t}$, *N* represents the length of $X_{t}$ and *k* is called the time lag.

**Figure 5:**
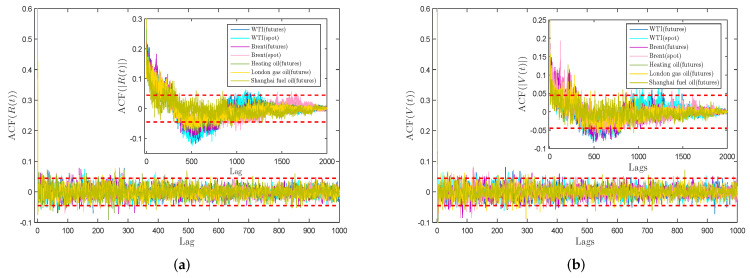
(**a**) Autocorrelation functions of return series R(t); (**b**) Autocorrelation functions of VMPD series V(t).

Figure 5 displays the autocorrelation functions of R(t) and V(t) of energy data sets related to distinct time lags. The red dashed locates in each graph stand for the 95% confidence interval. For R(t) and V(t), the ACF values stay in the 95% confidence interval as the time lag grows, that is, the autocorrelation of each time series gradually disappears with the increase of time lag, which indicates that future data is independent of historical data for R(t) and V(t). The absence of autocorrelation for returns series R(t) at large time lag suggests that the weak-form Efficient Market Hypothesis (EMH) may support these energy markets in the long run, and investors may not be able to use the technical means to gain profit-making opportunities [56,57]. For |R(t)| and |V(t)|, the two inset graphs exhibit that the shapes of autocorrelation function are parallel. They firstly decay slowly, and then go up as the lag value increases. In the end, nearly all the ACF values are in the 95% confidence interval. This is consistent with the empirical fact that security markets do not exhibit any significant autocorrelation.

## 5. Complexity Synchronization of Return Series and VMPD Series

For purpose of measuring the synchrony of energy markets in a new way, a novel nonlinear method called the cross CID FuzzyEn (CCF) composed of cross-fuzzy entropy and complexity-invariant distance is firstly proposed in this paper. Implementing CCF analysis for seven representative energy items, synchronization properties of returns and VMPD series are compared and analyzed. Also, we introduce the ensemble empirical mode decomposition (EEMD) algorithm to resolve returns and VMPD series into the intrinsic mode functions, and the degree that they follow the properties of initial sequence is investigated.

### 5.1. Mathematical Description of CCF Analysis

Entropy has been widely applied as a measurement of complexity. Fuzzy entropy (FuzzyEn) [39,40] evolved from approximate entropy (ApEn) [38] and sample entropy (SampEn) [37]. It uses the exponential function and its shape to define the similarity of vectors by introducing the fuzzy set. A lot of work has shown that FuzzyEn is better than ApEn and SampEn on reflecting regularity and similarity of time sequence. Furthermore, Chen et al. [43] extended the FuzzyEn method to the cross-fuzzy entropy so as to compare two different time series and evaluate their pattern synchronization degree. The cross-fuzzy entropy is relatively consistent and less dependent on record length in measuring regularity and asynchrony of time series.

For two *N*-sample time series {x(t),t∈{1,2,⋯,N}} and {y(t),t∈{1,2,⋯,N}}, the cross-fuzzy entropy (C-FuzzyEn) is constructed as follows:

(a) Given the embedding dimension *m*, we can define *m*-dimensional sequence vectors $X_{m}\left(i\right)$ and $Y_{m}\left(i\right)$ as follows
  (5) $$X_{m}\left(i\right)=\left\{x\left(i\right),x\left(i+1\right),\cdots ,x\left(i+m-1\right)\right\}-x_{0}\left(i\right),\;1\leq i\leq N-m+1$$
  (6) $$Y_{m}\left(i\right)=\left\{y\left(i\right),y\left(i+1\right),\cdots ,y\left(i+m-1\right)\right\}-y_{0}\left(i\right),\;1\leq i\leq N-m+1$$
  where $x_{0}\left(i\right)$ and $y_{0}\left(i\right)$ denote the average of series segment, that is
  (7) $$x_{0}\left(i\right)=\frac{1}{m}\sum_{j=0}^{m-1}x\left(i+j\right),\;y_{0}\left(i\right)=\frac{1}{m}\sum_{j=0}^{m-1}y\left(i+j\right),\;1\leq i\leq N-m+1.$$
(b) $d_{m}\left(i,j\right)$ represents the distance between vector $X_{m}\left(i\right)$ and vector $Y_{m}\left(j\right)$
  (8) $$d_{m}\left(i,j\right)=d\left[X_{m}\left(i\right),Y_{m}\left(j\right)\right]=\max\{|x\left(i+k\right)-x_{0}\left(i\right)-\left(y\left(j+k\right)-y_{0}\left(j\right)\right)\left|,\;0\leq k\leq m-1\right\}.$$
(c) The degree of synchrony $D_{m}\left(i,j\right)$ can be defined by fuzzy function $\mu (d_{m}\left(i,j\right),n,r)$
  (9) $$D_{m}\left(i,j\right)=\mu \left(d_{m}\left(i,j\right),n,r\right)=e^{-{\left(d_{m}\left(i,j\right)\right)}^{n}/r}.$$
(d) For all 1≤i,j≤N−m+1, we work out the mean values of $D_{m}\left(i,j\right)$, given by $\phi_{m}\left(n,r\right)$
  (10) $$\phi_{m}\left(n,r\right)=\frac{1}{N-m}\sum_{i=1}^{N-m}\left(\frac{1}{N-m-1}\sum_{j=1,j\neq i}^{N-m}D_{m}\left(i,j\right)\right).$$
(e) Finally, we can calculate the C-FuzzyEn of two time series {x(t)} and {y(t)} as
  (11) $$\mathrm{C-FuzzyEn}\left(m,n,r\right)=\lim_{N\to \infty }\left(\ln\phi_{m}\left(n,r\right)-\ln\phi_{m+1}\left(n,r\right)\right).$$
(f) *N* is finite, then the C-FuzzyEn(m,n,r) can be obtained as
  (12) $$\mathrm{C-FuzzyEn}\left(m,n,r\right)=\ln\phi_{m}\left(n,r\right)-\ln\phi_{m+1}\left(n,r\right).$$
  The parameter *m* represents the embedding dimension, *n* is the gradient of boundary, and *r* is called as the width. In the following empirical analysis, *m* and *n* are set to be 2.

Complexity-invariant distance (CID) [46,47] has been widely applied in measuring complexity differences between two time sequences. The calculation steps are as follows: For two time series X={x(t),t=1,2,⋯,N} and Y={y(t),t=1,2,⋯,N} with the same length of *N*, their complexity-invariant distance is given by

(13) CID(X,Y)=ED(X,Y)×CF(X,Y)

where ED(X,Y) is the Euclidean distance of *X* and *Y*, which is calculated by

(14) $$\mathrm{ED}\left(X,Y\right)=\sqrt{\sum_{i=1}^{N}{\left[x(i)-y(t)\right]}^{2}}$$

and CF(X,Y) is a complexity correlation factor calculated by

(15) $$\mathrm{CF}\left(X,Y\right)=\frac{\max(\mathrm{CE}(X),\mathrm{CE}(Y))}{\min(\mathrm{CE}(X),\mathrm{CE}(Y))}.$$

CE(X) and CE(Y) are the complexity estimates for X={x(t)} and Y={y(t)} respectively, which are shown as follows

(16) $$\mathrm{CE}\left(X\right)=\sqrt{\sum_{i=1}^{N-1}{\left[x(i)-x(i+1)\right]}^{2}},\;\mathrm{CE}\left(Y\right)=\sqrt{\sum_{i=1}^{N-1}{\left[y(i)-y(i+1)\right]}^{2}}.$$

The cross CID FuzzyEn between {x(t)} and {y(t)} is defined by

(17) CCF(x,y)=C-FuzzyEn(x,y,m,n,r)×CID(x,y).

The corresponding correlation coefficient between {x(t)} and {y(t)} is given as

(18) CorrelationCoefficient(x,y)=exp{−CCF(x,y)}.

It is worth noting that the CCF value measures the synchronism between two time series of the same length. To sum up, the lesser the CCF value is, or the bigger the CCF correlation coefficient is, the more synchronized the two time sequences are.

### 5.2. Mathematical Description of Ensemble Empirical Mode Decomposition

Empirical mode decomposition (EMD) [48], is a fully adaptive method so as to study nonlinear and non-stationary properties of time series. A signal is decomposed step by step into fluctuations or trends of different scales (frequency), and then produce a series of intrinsic mode functions (IMFs) and a residual sequence. The residual sequence is monotonic or average, which can represent the long-term trend or average state of the initial time sequence, and IMFs represent the distinct scales and produce adaptive data. An IMF sequence has two characteristics: (i) The difference between the extreme points of the time series and the zero crossings is less than or equal to 1; (ii) At every moment, the mean of the maximum envelope (upper envelope) and the minimum envelope (lower envelope) must be zero.

EMD decomposition is prone to modal aliasing. The ensemble empirical mode decomposition (EEMD) is a noise-assisted data analysis method proposed for the shortcomings of the EMD method. Wu and Huang [49] introduces white noise into the signal to be analyzed. The spectrum of white noise is uniformly distributed, so that the signal is automatically distributed to the appropriate reference scale, which complements some missing scales and has good performance in signal decomposition. The algorithm steps of EEMD are as follows:

(a) Add a white noise sequence $n_{m}$ to the time series *x* in the *m*-th test
  (19) $$x_{m}\left(t\right)=x\left(t\right)+n_{m}\left(t\right).$$
(b) Use the same algorithm as traditional EMD [48] to resolve $x_{m}$ into IMFs $c_{j,m}$ and residue *r*
  (20) $$x_{m}\left(t\right)\to \sum_{j=1}^{n}c_{j,m}\left(t\right)+r\left(t\right).$$
(c) Repeat Procedure (a) and Procedure (b) for a pre-set value *M* of tests, and apply distinct white noise sequences which have the same amplitude every time.
(d) Work out the mean value as the final IMFs, which is given by
  (21) $$IMF_{j}\left(t\right)=\frac{1}{M}\sum_{m=1}^{M}c_{j,m},\;j=1,2,\cdots ,p,\;\;m=1,2,\cdots ,M.$$

**Figure 6:**
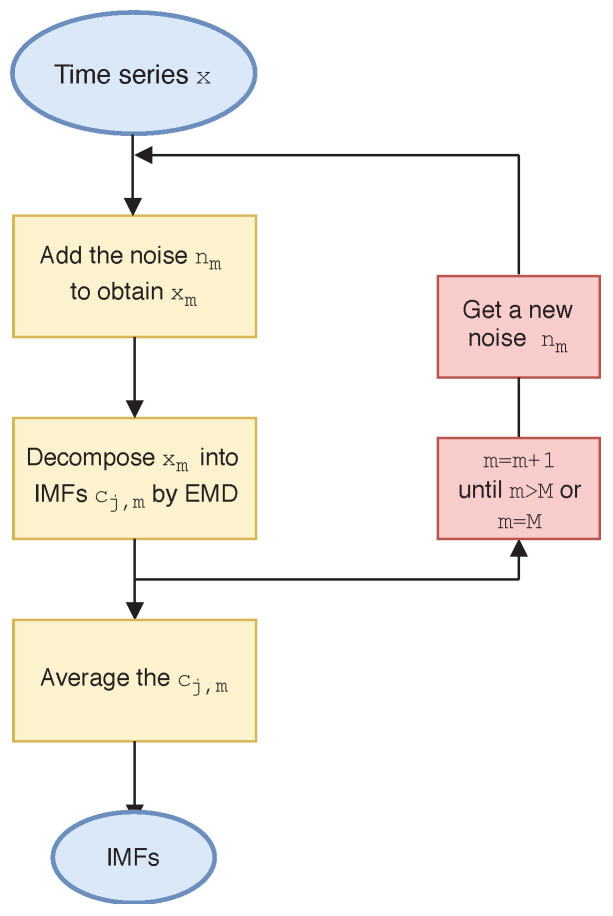
Flow chart of ensemble empirical mode decomposition algorithm.

**Figure 7:**
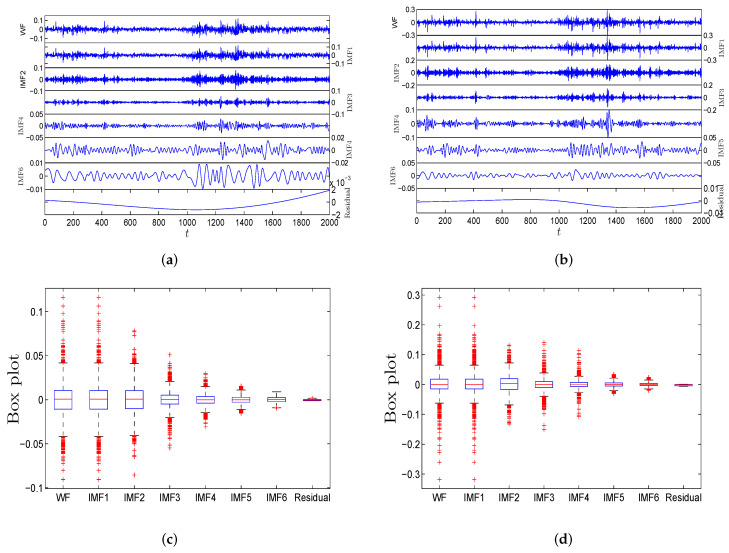
(**a**) Decomposition results of R(t) for WF with five separate IMFs by EEMD; (**b**) Decomposition results of V(t) for WTI futures with five separate IMFs by EEMD; (**c**) Box plots of five separate IMFs of R(t) for WF; (**d**) Box plots of five separate IMFs of V(t) for WF.

In order to better illustrate the above algorithm, Figure 6 shows the flow chart of the EEMD method. Figure 7a,b show the decomposition results of R(t) and V(t) of WF by the EEMD algorithm. The plots show five IMFs (IMF1 to IMF5) and one residual. Figure 7c,d show the box plots of five IMFs to represent the basic statistical information, which clearly indicate that the fluctuation scale of IMF1 to IMF5 decreases successively.

### 5.3. Empirical Study for Complexity Synchronization

**Figure 8:**
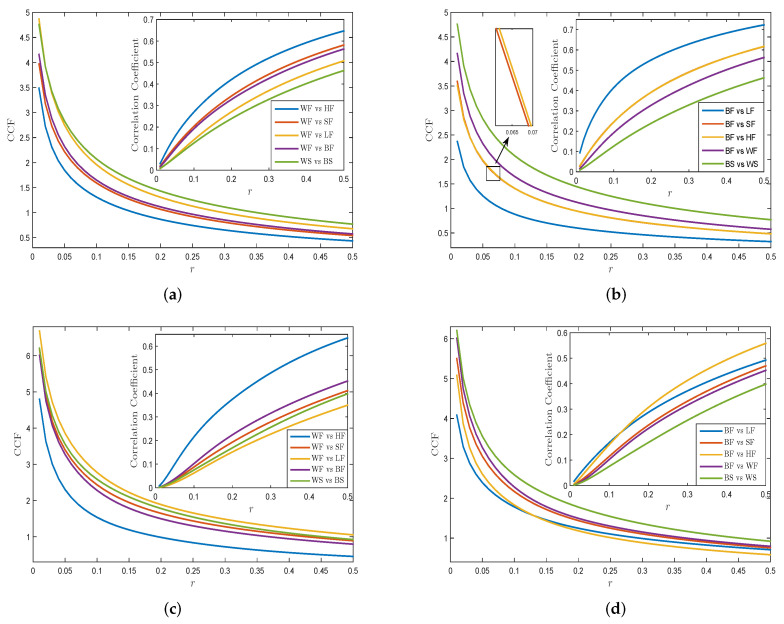
(**a**) CCF values of WF returns and WS returns with other energy items; (**b**) CCF values of BF returns and BS returns with other energy items; (**c**) CCF values of WF and WS for VMPD series with other energy items; (**d**) CCF values WF and WS for VMPD series with other energy items.

We apply the CCF approach to study the complexity synchronization feature of returns and the VMPD series for different energy items. WTI crude oil and Brent crude oil are chosen as references for comparison with other energy data. Figure 8a,c show the graphs of CCF values and correlation coefficient of returns and VMPD series for WF and WS with other energy items related to different values of *r*, where m=2, n=2 and *r* is set from 0 to 0.5. From Figure 8a, we observe that the figure of WF returns with HF returns remains in the lowest part, while the curves for WF returns with futures of other exchanges stay in the higher part. This shows that the synchronization of WF returns with the futures return series of its own market is higher than that of WF returns with the futures return series of other market. It is worth noting that the CCF curve between WF and BF is located below that between WS and BS, which indicates that for the WTI crude oil and the Brent crude oil, the synchronization between futures returns is greater than that between spot returns. The results in Figure 8c are similar to those in Figure 8a. For all the VMPD series, we can see that CCF values reduce as the parameter *r* rises, and tend to be stable in the end, that are semblable to the results of return sequence. In Figure 8c, the curve of WF with HF for VMPD series also remains in the lowest part. We can also conclude from Figure 8c that for VMPD sequence, the WF is the most synchronized with the HF that belongs to the New York Mercantile Exchange, and the WF has the weakest synchronization with the LF that belongs to the Intercontinental Exchange.

Figure 8b,d demonstrate the plots of CCF values and correlation coefficients of returns and VMPD series for BF and BS with other energy items for different values of *r*. In Figure 8b, the curve of BF returns with LF returns remains in the lowest part, and the curve of BF returns with SF returns stays in the middle. And different energy return series appear obvious clustering behaviors in the CCF method graphs in terms of their markets. We may draw a conclusion that BF returns has the strongest synchronization with LF returns which belong to the same exchange as BF. The general trend and spatial distribution of each curve in Figure 8d is roughly the same as the curve in Figure 8b. As for the VMPD series of BF, the distance between each curve in Figure 8d is closer, and as the value of *r* increases, the gap between them decreases, and finally they are almost intertwined. Table 3 and Table 4 exhibit the CCF correlation coefficients of each returns and VMPD series pairs, respectively. It can be seen that nearly each correlation coefficient of VMPD sequences in Table 4 is smaller than those of returns in Table 3, which indicates a weaker correlation between the VMPD sequences.

**Figure 9:**
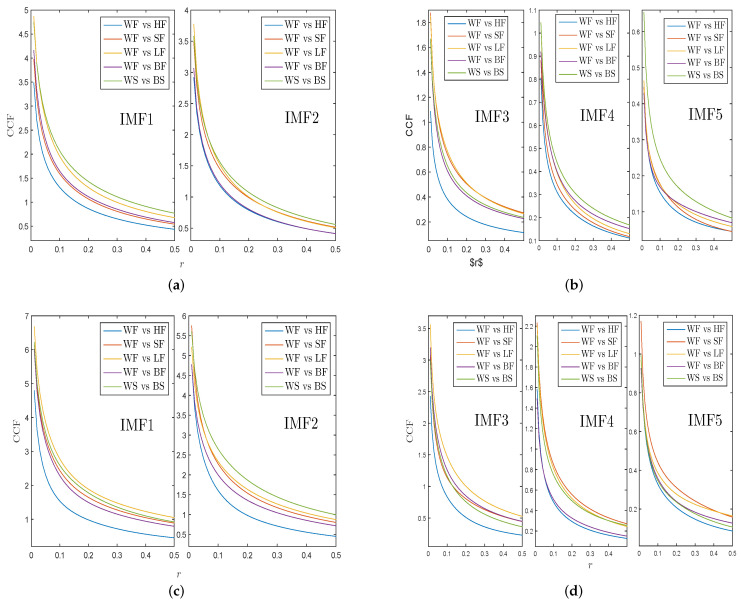
(**a**) CCF values of IMF1-IMF2 for WF returns and WS returns with other energy items; (**b**) CCF values of IMF3-IMF5 for WF returns and WS returns with other energy items; (**c**) CCF values of IMF1-IMF2 of WF and WS for VMPD series with other energy items; (**d**) CCF values of IMF3-IMF5 of WF and WS for VMPD series with other energy items.

Next, we decompose returns and VMPD sequences of different energy data into corresponding IMFs by applying the EEMD algorithm to explore whether IMFs have analogical complexity synchronization behaviors as initial security time sequence. Figure 9a,b exhibit the CCF values of IMFs of WF returns and WS returns with other energy items. Figure 9c,d show the CCF values of IMFs of WF and WS for VMPD series with other energy items. IMF*i* (i=1,2,⋯,5) represents the *i*th IMF. In Figure 9, all the curves are smoother and have almost the same order as the curves in Figure 8a,c. This indicates that IMF1 and IMF2 retain most of statistical characteristics of the initial time sequence. In general, the IMFs obtained by the EEMD method partly have the complexity synchronization properties of the initial sequence, while the IMF1 and IMF2 sequences retain most information about the original sequence.

## 6. Conclusions

The study of return volatility for energy markets is extremely important in quantifying ventures and optimizing portfolios. The volatility monotonous persistence duration (VMPD) time sequence is firstly introduced into the research of energy markets to describe the security fluctuation behaviors from a new perspective. We adopt seven kinds of energy data including futures and spot from various markets, and the statistical properties and complex synchronization behaviors are studied. The price formation of economic market is the result of the complicated action of many factors, and entropy is an index of pattern diversity in time series. Based on fuzzy entropy and complex invariant distance, we construct a new method to measure complexity synchronization of VMPD series and return series for energy futures and spot. The main conclusions of this paper are as follows.

The VMPD sequence unites the maximum fluctuation difference and the continuous variation length, which is regarded as a novel indicator to evaluate investment risks. The range of changes in energy futures and spot prices may affect the investment attitudes of market participants, as drastic price changes may bring investment risks, thus making some market traders tend to adopt a more conservative attitude. Since VMPD quantifies the intensity of continuous volatility series, it can provide investors with short-term volatility trend, which is of great significance for investors to judge the risk level in the future.

Further, we observe two important statistic and nonlinear characteristics for the energy VMPD sequences: probability distribution and autocorrelation behavior. Empirical results show that return series and the VMPD series for the energy items exhibit excess kurtosis, fat-tail distributions, and absence of autocorrelation, specifically, the probability distribution is very well drawn by the power-law function.

Moreover, a new method called the cross CID FuzzyEn composed of cross-fuzzy entropy and complexity-invariant distance is firstly proposed in this paper. Implementing CCF analysis for seven representative energy items, the synchronization properties of returns and VMPD series are comparatively studied. It is shown that both return series and VMPD sequence gradually appear more synchronous with the width *r* increasing, and various energy return series present obvious clustering behavior in accordance with markets.The synchronization between futures series is greater than that between spot series, for WTI crude oil and Brent crude oil.

Then we apply the ensemble empirical mode decomposition (EEMD) to resolve returns and VMPD sequence into the intrinsic mode functions, and the degree that they follow the synchronization features of initial sequence is investigated. The empirical analysis reveals that the resolved data maintain some statistical and linear properties of initial energy time sequences.

All in all, based on the new volatility statistics and the proposed method of measuring complex synchronicity, we have done a large number of statistical and complex analyses of oil futures and spot data in several energy markets. As an emerging energy market, China energy futures market, just like mature energy futures markets in Europe and America, is a complex nonlinear system, and traditional theories and methods cannot effectively reflect the complex nonlinear dynamics characteristics. This paper provides a new perspective to describe volatility behaviors of energy markets, which is expected to be a new indicator to avoid risks. Compared with the return series, VMPD sequence has both commonness and difference, and it can describe the volatility behavior of energy futures and spot from a new angle, which is conducive to quantifying risks. For China, an important oil importer, it is necessary to establish a sound oil futures and spot market. This paper aims to provide a new reference for the managers and participants in the securities market. In terms of energy portfolio risks, in the short term, we should focus on the extent of continuous price rise (or fall) and continuous fluctuation duration in the energy futures and spot markets, so as to prevent and deal with the risk price fluctuation more effectively. Specifically, the significance lies in two aspects. Firstly, VMPD can reasonably describe the market fluctuations, guide investors to conduct optimal investment behavior, help investors avoid market risks, and obtain direct economic benefits. Secondly, it can provide a new perspective for financial researchers to explore energy market fluctuations, so as to provide some desirable guidance for the establishment of national macroeconomic policies. It can be seen that the research in this paper can not only bring practical economic significance and value, but also meet the development needs of social and economic construction.

In the process of measuring the reliability of the newly proposed statistics, we proposed a new method to measure the complexity synchronization. The new CCF method is valuable and can be applied to many aspects of fields, such as the comparison of different market products and the relationship of oil price and stock price. The new concept of VMPD series and the novel approach of measuring complexity synchronization can make the research on the fluctuation behaviors of energy markets be more abundant to a certain extent. 

## Figures and Tables

**Table 1:** Descriptive statistics and K-S test for return and VMPD series of energy data.

		Descriptive Statistics							K-S Test	
Returns		Mean	Std	Min	Max	Kurtosis	Skewness		stat	*H*
WF		−0.0001	0.0204	−0.0907	0.1162	5.8938	0.1460		0.4715	1.0000
WS		−0.0001	0.0198	−0.1065	0.1172	6.0158	0.0262		0.4710	1.0000
BF		−0.0001	0.0190	−0.0896	0.1042	6.1393	0.1108		0.4711	1.0000
BS		−0.0001	0.0195	−0.0825	0.0990	5.4787	0.2114		0.4737	1.0000
HF		0.0000	0.0178	−0.1973	0.1038	13.3248	−0.6039		0.4729	1.0000
LF		−0.0000	0.0161	−0.0899	0.1209	7.8970	0.3297		0.4753	1.0000
SF		0.0004	0.0184	−0.2480	0.1215	22.9517	−1.1360		0.4742	1.0000
**VMPD**		**Mean**	**Std**	**Min**	**Max**	**Kurtosis**	**Skewness**		**stat**	***H***
WF		0.0002	0.0404	−0.3188	0.2920	11.2956	−0.3545		0.4462	1.0000
WS		0.0004	0.0374	−0.3128	0.2250	12.1775	−0.8374		0.4510	1.0000
BF		0.0017	0.0394	−0.3240	0.2644	11.4187	−0.2208		0.4446	1.0000
BS		0.0017	0.0400	−0.2844	0.3496	15.6557	0.7868		0.4491	1.0000
HF		0.0007	0.0397	−0.7714	0.3642	81.2650	−3.3956		0.4513	1.0000
LF		0.0005	0.0327	−0.2136	0.2296	10.3138	−0.0179		0.4535	1.0000
SF		−0.0003	0.0387	−0.4821	0.3376	24.3661	−0.8844		0.4473	1.0000

**Table 2:** Estimation values of γ and β of the last 10% part of |R(t)| and |V(t)| series.

**Absolute Returns**	γ		β		$R^{2}$
WF	−3.5225	[−3.6036, −3.4415]	−14.1690	[−14.4209, −13.9170]	0.9742
WS	−3.4647	[−3.5343, −3.3950]	−14.1205	[−14.3397, −13.9014]	0.9802
BF	−3.3820	[−3.4449, −3.3191]	−13.9679	[−14.1679, −13.7679]	0.9830
BS	−3.7610	[−3.8267, −3.6953]	−15.0741	[−15.2810, −14.8673]	0.9852
HF	−3.1425	[−3.2046, −3.0804]	−13.5066	[−13.7100, −13.3032]	0.9807
LF	−3.2734	[−3.3305, −3.2164]	−14.2189	[−14.4090, −14.0288]	0.9861
SF	−4.1244	[−4.2191, −4.0296]	−16.2797	[−16.5734, −15.9860]	0.9809
**Absolute VMPD**	γ		β		$R^{2}$
WF	−2.8520	[−2.9075, −2.7966]	−10.0813	[−10.2152, −9.9474]	0.9814
WS	−3.0446	[−3.0999, −2.9894]	−10.7910	[−10.9287, −10.6533]	0.9838
BF	−2.8119	[−2.9011, −2.7227]	−9.9982	[−10.2141, −9.7823]	0.9517
BS	−2.6108	[−2.6395, −2.5820]	−9.6324	[−9.7034, −9.5615]	0.9940
HF	−2.6961	[−2.7462, −2.6460]	−10.0468	[−10.1742, −9.9195]	0.9829
LF	−2.6410	[−2.6972, −2.5847]	−10.1502	[−10.2981, −10.0023]	0.9780
SF	−2.8223	[−2.8821, −2.7624]	−10.1431	[−10.2863, −9.9998]	0.9819

**Table 3:** CCF correlation coefficients of two return series for different values of *r*.

Rerurns	r=0.1	r=0.2	r=0.3	r=0.4	r=0.5
BF vs. LF	0.4143	0.5508	0.6289	0.6832	0.7234
BF vs. SF	0.2449	0.3919	0.4906	0.5620	0.6168
BF vs. HF	0.2445	0.3918	0.4897	0.5618	0.6175
BF vs. WF	0.1922	0.3280	0.4258	0.5022	0.5627
BS vs. WS	0.1261	0.2391	0.3289	0.4021	0.4628
WF vs. HF	0.2699	0.4209	0.5206	0.5923	0.6468
WF vs. SF	0.2036	0.3441	0.4457	0.5219	0.5812
WF vs. LF	0.1411	0.2706	0.3694	0.4465	0.5077
WF vs. BF	0.1922	0.3280	0.4258	0.5022	0.5627
WS vs. BS	0.1261	0.2391	0.3289	0.4021	0.4628

**Table 4:** CCF correlation coefficients of two VMPD series for different values of *r*.

VMPD	r=0.1	r=0.2	r=0.3	r=0.4	r=0.5
BF vs. LF	0.1685	0.2873	0.3719	0.4381	0.4924
BF vs. SF	0.1146	0.2371	0.3314	0.4072	0.4699
BF vs. HF	0.1606	0.3067	0.4128	0.4940	0.5583
BF vs. WF	0.1024	0.2242	0.3159	0.3901	0.4523
BS vs. WS	0.1024	0.2242	0.3159	0.3901	0.4523
WF vs. HF	0.2157	0.3750	0.4866	0.5710	0.6349
WF vs. SF	0.0879	0.1932	0.2793	0.3508	0.4114
WF vs. LF	0.0624	0.1515	0.2274	0.2924	0.3497
WF vs. BF	0.1024	0.2242	0.3159	0.3901	0.4523
WS vs. BS	0.0754	0.1690	0.2564	0.3329	0.3982

## Abbreviations

The following abbreviations are used in this manuscript:

VMPD	Volatility monotonous persistence duration		WF	Crude oil WTI futures
C-FuzzyEn	Cross-fuzzy entropy		WS	Crude oil WTI spot
CID	Complexity invariant distance		HF	Heating oil futures
CCF	Cross complexity invariant distance FuzzyEn		BF	Crude oil Brent futures
IMF	Intrinsic mode function		BS	Crude oil Brent spot
EMD	Eempirical mode decomposition		LF	London gas oil futures
EEMD	Ensemble empirical mode decomposition		SF	Shanghai fuel oil futures
